# The Refraction of the Eye

**Published:** 1901-03

**Authors:** 


**The Refraction of the Eye.** By A. EDWARD DAVIS, M.D. Pp. xii., 431. New York: The Macmillan Company. 1900.

This book deals chiefly with the practical value of the ophthalmometer of Javal and Schiötz in examining the refraction of the eye, and does not to any extent enter into the mathematical part of the subject. In it the author endeavours to reproduce as far as is possible in a book the instruction given at the New York Post-Graduate Medical School, where the teaching is entirely clinical. The construction of the instrument and the principles upon which it is based are explained, clear rules for its use are given, and the author's high opinion as to its value in detecting the presence of astigmatism and estimating its degree is expressed. The various forms of ametropia are dealt with at great length, and their treatment is illustrated by very numerous examples, special mention being made of the help to be gained from the use of the ophthalmometer in cases of irregular astigmatism and keratoconus.

A chapter is devoted to strabismus and insufficiency of the ocular muscles, in which the etiology of these affections and the methods of testing them are well but not exhaustively described.

The book, although somewhat redundant in parts, is of a thoroughly practical character, and can be recommended to anyone wishing to obtain a knowledge of the uses and limits of the ophthalmometer.

